# Whole-Genome Sequencing Identified *KCNJ12* and *SLC25A5* Mutations in Port-Wine Stains

**DOI:** 10.3389/fmed.2022.905902

**Published:** 2022-07-20

**Authors:** Kai Chen, Yan-Yan Hu, Lin-Lin Wang, Yun Xia, Qian Jiang, Lan Sun, Shan-Shan Qian, Jin-Zhao Wu, Liu-Qing Chen, Dong-Sheng Li

**Affiliations:** ^1^Hubei Key Laboratory of Infectious and Immune Skin Diseases, Wuhan No. 1 Hospital, Tongji Medical College, Huazhong University of Science and Technology, Wuhan, China; ^2^Department of Dermatology, Wuhan No. 1 Hospital, Tongji Medical College, Huazhong University of Science and Technology, Wuhan, China; ^3^School of Medicine, Jianghan University, Wuhan, China

**Keywords:** whole genome sequencing (WGS), port-wine stains (PWS), *KCNJ12*, *SLC25A5*, mutation

## Abstract

Port-wine stains (PWSs) are a congenital capillary malformed disorder and are caused by a number of somatic mutations that disrupt vascular development. However, the underlying genetic mutations in the pathogenesis of PWS have not yet been fully elucidated. To understand PWS genetic variations and investigate novel genetic mutations, we extracted genomic DNA from four sporadic PWS patients and then performed whole-genome sequencing (WGS). Using Sorting Intolerant from Tolerant (SIFT), PolyPhen2, Mutation Assessor, MetaSVM to identify candidate genetic mutations and whole-exome sequencing (WES) to confirm the identified variants. We found a previously reported G protein subunit alpha q (GNAQ) mutation c.548G > A, p.Arg183Gln in one case, whereas no such mutation was found in the other three samples. Moreover, six novel somatic mutations in three genes, including *KCNJ12*, *SLC25A5*, *POTEE*, were found in these four samples. Importantly, WES also verified the *KCNJ12* (c.433G > A, p.Gly145Ser) and *SLC25A5* (c.413G > A, p.Arg138His) mutations in other five sporadic PWS patients, with the frequency of 60% (3 of 5) and 40% (2 of 5), respectively. Thus, we reveal in this study two novel somatic mutations, *KCNJ12* and *SLC25A5*, in the sporadic PWS patients for the first time. These findings highlight the genetic polymorphism of PWS and provide potential clinical prediction targets for this disease.

## Introduction

Port-wine stain (PWS) is a kind of congenital capillary malformation that often occur on the face and neck, with an incidence of 3–5/1000 in newborn worldwide (1). Because of its sporadic with no heritability, a long-standing hypothesis suggests that PWS are associated with somatic mutations (2). In 2013, Shirley and colleagues performed whole-genome sequencing (WGS) of DNA from 13 patients with non-syndromic PWS, discovered a non-synonymous mutation (c.548G > A, p.Arg183Gln) in the G protein subunit alpha q (*GNAQ*) gene, and defined this gene as a causative mutation of PWS (3). A later finding also confirmed the presence of somatic *GNAQ* mutation in a sporadic PWS, as well as novel somatic mutations in several other genes (4). Similarly, next-generation sequencing data revealed the different *GNAQ* mutation frequencies in the structures of patients with PWS (5). Although the genetic mutation of *GNAQ* is important in the pathogenesis of PWS, not all PWS patients have a mutation in this gene, suggesting other genetic etiologies could also contribute to the pathologic manifestations of this disorder.

KCNJ12 (also known as IRK2, KCNJN1, and Kir2.2) is a Kir ion channel protein, which encodes potassium inwardly-rectifying channel 12 and contributes to the cardiac inward rectifier current (IK1). This protein is thought to be involved in protein homotetramerization and regulation of resting membrane potential (6). In skin development, numerous studies have shown the important roles of *KCNJ12* mutations in skin cancers, such as colorectal carcinoma (CRC), head and neck squamous cell carcinoma (HNSCC), and esophageal squamosa cell carcinoma (ESCC) (7–9). Moreover, *KCNJ12* mutation also was identified in ultraviolet b (UVB)-irradiated primary human keratinocytes (10). Taken together, these studies indicated the vital roles of *KCNJ12* mutations in skin tumorigenesis. However, the presence of *KCNJ12* mutations in PWS has not been previously reported.

Solute carrier family 25 member 5 (SLC25A5) is a member of the mitochondrial solute carrier subfamily protein and the function of this protein remains unclear, whereas *SLC25A5* mutation has been reported in the samples from sporadic Alzheimer’s disease (AD) patients (11, 12). To date, no study concerning the connection between *SLC25A5* mutation and PWS pathogenesis. In this study, by using WGS technology, we identified two novel genetic mutations in *KCNJ12* and *SLC25A5* genes in sporadic PWS patients, and the genetic variant of these two genes was validated by the whole-exome sequencing results. Identifying these somatic mutations of PWS may provide insight into the clinical prediction of this disease.

## Methods

### Study Approval

Each patient signed informed consent for study participation and clinical images and publication of identified information prior to the study. All the sequencing was approved by the ethics committee of Wuhan No. 1 Hospital affiliated with Tongji Medical College, Huazhong University of Science and Technology.

### Images Taken

Images of the skin lesions of each patient were taken using the Canon 650D SLR camera. Dermoscopic and reflectance confocal microscopy (RCM) images were taken using the ATBM FotoFinder Bodystudio dermatoscope and VivaScope. Functional magnetic resonance imaging (MRI) was scanned using the Siemens Magnetom Verio 3.0T.

### Hematoxylin-Eosin Staining

Port-wine stain lesional tissue was embedded in paraffin and cut into sections as described previously (13). The sections were stained with HE and observed under an Automated Digital Slice Scanning System (ZEISS, Axioscan 7).

### DNA Extraction

Genomic DNA was extracted from PWS lesional tissue as previously described (5). DNA concentrations and quality were determined using a Multiskan Spectrum (SpectraMax 190, Thermo Scientific, Wilmington, United States).

### Whole-Genome Sequencing/Whole-Exome Sequencing and Bioinformatic Analysis

Whole-genome sequencing/whole-exome sequencing was performed by Beijing Genomics Institute (BGI)-Shenzhen Biotechnology Company (Shenzhen, China). The samples were treated according to the method previously reported and pair-end 100 base reads were generated in the way of sequenced combinatorial Probe-Anchor Synthesis (cPAS) on BGISEQ-500 platform (14). The raw sequencing data were processed using the following steps: (1) Removing reads containing sequencing adapter; (2) Removing reads whose low-quality base ratio (base quality less than or equal to 5) is more than 50%; (3) Removing reads whose unknown base (‘N’ base) ratio is more than 10%. Statistical analysis of data and downstream bioinformatics analysis were performed on this filtered, high-quality data, referred to as the clean data. Clean data were aligned to the human reference genome using Burrows-Wheeler Aligner (BWA) (15). Picard was used to remove duplicated sequence reads. Realignment was performed with the Genome Analysis Toolkit (GATK) (16). Genetic variations were identified using HaplotypeCaller of GATK and annotated with SnpEff software (17). All candidate variants were filtered against public databases including the 1,000 Genomes Project, and the Single Nucleotide Polymorphism Database (dbSNP). The prediction tools, Sorting Intolerant from Tolerant (SIFT), PolyPhen2, Mutation Assessor, and MetaSVM were used to estimate the likelihood that an amino acid transition may affect the function of the protein. The mutation site that presents in at least two samples was chosen for the potential genetic variant. Variants were visualized using the Integrative Genomic Viewer (IGV).

## Results

### Clinical Characteristics

**FIGURE 1:**
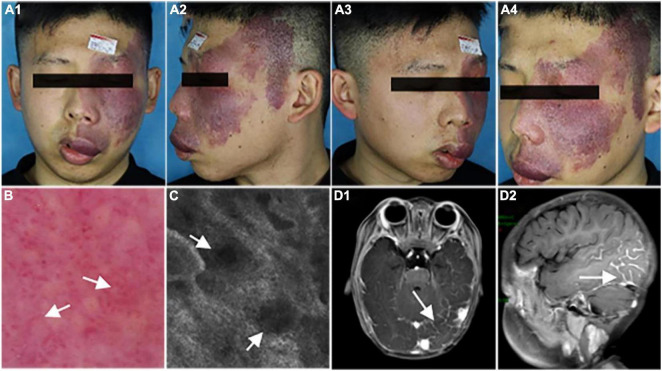
Representative clinical manifestations, dermoscopy finds, reflectance confocal microscopy (RCM) images, and magnetic resonance imaging (MRI) scans of the patient. **(A1–A4)** Port-wine stain (PWS) on the central and lateral face of the patient. **(B,C)** Representative dermoscopy and RCM pictures of the PWS lesions from the patient. White arrows indicate the expansive vessels. **(D)** Representative MRI scans of the patient. White arrows indicate an intracranial malformation in his brain tissue.

We show a representative case of a 22-year-old male patient (case 1) with a hypertrophic PWS on the left central face and lateral face (Figures 1A1–A4). The vascular morphology, including linear vessels, dots or globular vessels, and sausage-like vessels were seen in the dermoscopic picture (Figure 1B). Consistently, the RCM image indicated increased expansive blood vessels in the dermis (Figure 1C) and an MRI scan revealed obvious intracranial malformations in his brain region (Figures 1D1,D2).

**FIGURE 2:**
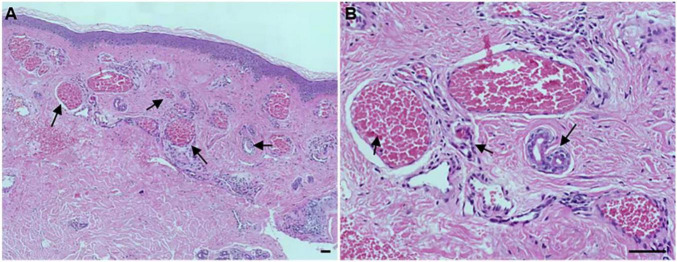
Histopathological examination of the port-wine stains (PWS) lesional tissue. Hematoxylin-eosin (HE)-stained PWS lesional tissue section showing ectatic capillaries. Black arrows indicate the expansive blood vessels in the dermis and filled erythrocytes in the lumen. **(A)**: original magnification × 5. **(B)**: original magnification × 20. Scale bars: 50 μm.

To determine the histological as well as the clinical symptoms changes, we performed a hematoxylin-eosin (HE) of the PWS lesional tissue section of this patient. Under the microscope, hyperplastic capillaries were found in the dermis (Figure 2A, HE, 5x), and ectatic vessels also were observed (Figure 2B, HE, 20x). Taken together, these results indicate that this patient is characterized by typical clinical features of PWS.

### Whole-Genome Sequencing and Somatic Variants Identification

In order to investigate the genetic variations in the PWS patient, we sequenced the whole genomes of lesional DNA samples from four sporadic PWS patients (case 1 to case 4). The demographic data and PWS features of each patient are described in Table 1. We detected a large number of somatic single nucleotide variations (SNVs) in all the samples across the chromosomes. SIFT, PolyPhen2, Mutation Assessor, and MetaSVM were used to identify pathogenic variants (18). After scoring (Mutation Assessor >3.5, MetaSVM >0, PolyPhen2 > = 0.909, SIFT < = 0.05), a previously reported GNAQ mutation c.548G > A, p.Arg183Gln was identified in one sample (case 1), whereas no such mutation was found in other three samples. Additionally, a total of six somatic variations in three genes, including *KCNJ12*, *SLC25A5*, *POTEE*, were found in these four sporadic PWS patients. The minor allele frequency (MAF) of each variant in the 1,000 Genomes Project also was listed in Table 2.

**TABLE 1:** Demographic data and port-wine stain (PWS) features of the patients in this study.

Case	Gender	Age (years)	PWS color	Localization	Side
1	Male	22	Hypertrophic	Central face/Lateral face	L
2	Female	33	Hypertrophic	Face/Neck	R
3	Male	24	Purple	Central face	R
4	Female	12	Red	Face	L/R
5	Female	2	Red	Face	L/R
6	Male	24	Purple	Face	R
7	Male	19	Hypertrophic	Lateral face/Neck	R
8	Female	2	Red	Face	L/R
9	Female	16	Hypertrophic	Lateral face	L

*L, left; R, right.*

**TABLE 2:** Whole-genome sequencing identified G protein subunit alpha q (*GNAQ*) and other novel somatic mutations in our patients.

Gene	Mutation	SNP	SIFT	PolyPhen2	Mutation assessor	Meta SVM	MAF
*GNAQ*	c.548G > A, p.Arg183Gln	rs397514698	0.0 (D)	0.936 (D)	3.655 (H)	1.0391 (D)	0.05
*KCNJ12*	c.415G > A, p.Glu139Lys	rs76265595	0.005 (D)	1.0 (D)	4.085 (H)	1.0984 (D)	0.49965
*SLC25A5*	c.217G > A, p.Gly73Ser	rs143413528	0.0 (D)	0.984 (D)	4.12 (H)	1.0179 (D)	0.38386
*SLC25A5*	c.707G > C, p.Arg236Pro	rs114413582	0.013 (D)	0.995 (D)	4.665 (H)	0.9409 (D)	0.1539
*POTEE*	c.2918G > A, p.Gly973Asp	rs62178369	0.0 (D)	1.0 (D)	4.33 (H)	0.9029 (D)	0.21757
*KCNJ12*	c.433G > A, p.Gly145Ser	rs75029097	0.005 (D)	1.0 (D)	4.1 (H)	0.8995 (D)	0.01999
*SLC25A5*	c.413G > A, p.Arg138His	rs200550329	0.032 (D)	0.979 (D)	3.86 (H)	0.7781 (D)	0.079

*SNP, single nucleotide polymorphism; SIFT, sorting intolerant from tolerant; PolyPhen, Polymorphism Phenotyping; SVM, support vector machine; D, damaging; H, high; MAF, minor allele frequency.*

### Whole-Exome Sequencing of *KCNJ12* and *SLC25A5* Variants in Other Five Port-Wine Stain Patients

**FIGURE 3:**
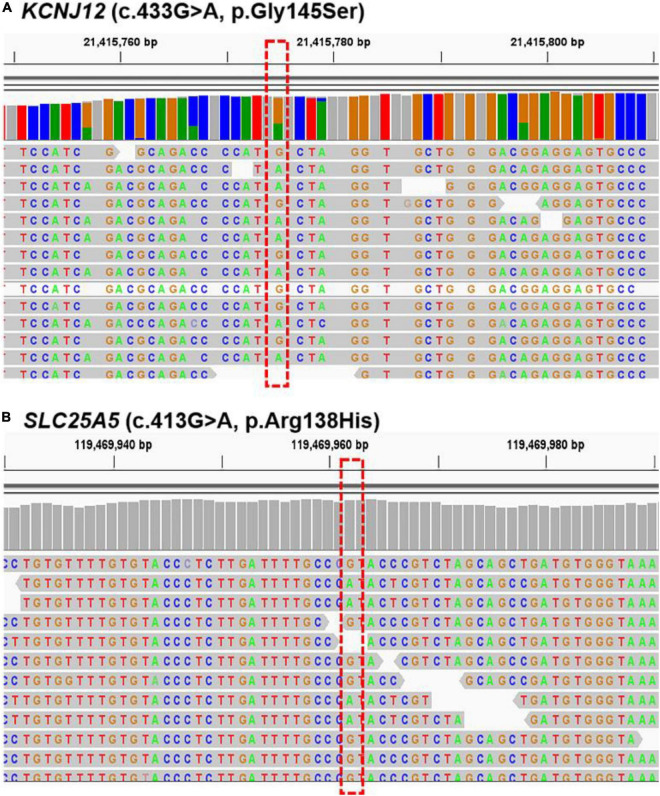
Sequencing results for *KCNJ12* and *SLC25A5* variants. Whole-exome sequencing (WES) data with the respective somatic *KCNJ12* mutation **(A)** and *SLC25A5* mutation **(B)** are shown from cases 8 and 9, respectively. The red dotted box indicated the mutation site.

We further verified the presence of these six targeted variant sites in the other five sporadic PWS patients (case 5 to case 9) *via* WES. The two mutations, *KCNJ12* (c.433G > A, p.Gly145Ser) and *SLC25A5* (c.413G > A, p.Arg138His), were validated in the patients (Figure 3), and we found the genetic variants in 60% (3 of 5) and 40% (2 of 5) of patients (Table 3), whereas no other genetic variants of those mentioned above were found in these five patients. Importantly, these two mutations were not detected in the samples from peripheral blood and matched control tissues. Therefore, *KCNJ12* (c.433G > A, p.Gly145Ser) and *SLC25A5* (c.413G > A, p.Arg138His) appear to be the causative somatic mutations of this disorder.

**TABLE 3:** Whole-exome sequencing verified the *KCNJ12* and *SLC25A5* variants in other patients.

Somatic mutations	Case 1	Case 2	Case 3	Case 4	Case 5	Case 6	Case 7	Case 8	Case 9	Frequency
*KCNJ12* (c.433G > A, p.Gly145Ser)	N	Y	Y	Y	Y	Y	N	Y	N	6/9
*SLC25A5* (c.413G > A, p.Arg138His)	N	N	Y	Y	N	Y	N	N	Y	4/9

*N, no; Y, yes.*

## Discussion

In this study, we performed WGS to identify novel genetic mutations in sporadic PWS patients. Our data suggested an association between *SLC25A5* mutation and increased risk of PWS. Consistently, a somatic heterozygous mutation in *SLC26A4* was found in a large vestibular aqueduct syndrome (LVAS)-associated PWS patient (19). This finding strongly implies that solute carrier protein plays a pivotal role during the progression of PWS. However, the underlying mechanisms remain unknown. As reported, phosphatidylinositol 3-kinase (PI3K) and protein kinase B (AKT) were activated in blood vessels of hypertrophic PWS and participated in nodule formation (20, 21). Moreover, long non-coding RNA SLC25A5-AS1 also has been functionally implicated in controlling the PI3K/AKT pathway in gastric cancer and carcinogenesis (22, 23). Thus, our data not only presented a novel genetic variation of *SLC25A5* gene in the pathogenesis of PWS but also suggested a potential regulatory signaling pathway by controlling PI3K/AKT activation.

Over the past years, mutations of *KCNJ12* have been found mainly in cardiovascular diseases and skin cancers (7, 24). Here, we firstly reported a novel genetic variation in the *KCNJ12* gene in PWS, but the causative mechanism was still elusive. It has also been reported that topical agents can suppress pulsed dye laser (PDL)-induced angiogenesis in the skin *via* blocking multiple signaling pathways, such as PI3K/AKT/mammalian target of rapamycin (mTOR) and extracellular regulated kinase (ERK) pathway (25). *RASA1*, another published mutation gene in PWS, also was found to regulate vascular development *via* inhibiting the AKT pathway (26). Thus, these findings suggest a critical role for the AKT pathway in the pathophysiological process of regeneration and revascularization of blood vessels in PWS. Interestingly, a recent study reported that KCNJ12 acts as a straightforward target of miR-132-3p to modulate the AKT signaling pathway in bladder cancer oncogenesis and metastasis (27). Therefore, it is possible that *KCNJ12* mutation caused PWS pathology by activating the AKT pathway, targeting KCNJ12 or the AKT signaling pathway might be a candidate intervention in the treatment of this disease.

Port-wine stains are a congenital vascular malformation and approximately 15–20% of the infant with ophthalmic (V1) trigeminal PWS are at risk of Sturge-Weber syndrome (SWS), a neurocutaneous disorder characterized by seizures, headaches, stroke, developmental delay, cognitive defects, and intellectual impairments (26, 28). Happle hypothesized that SWS and PWS have the same mutations, with the clinical manifestations dependent on where and when during development the somatic mutation occurs (2). In this study, our results confirmed the presence of two novel genetic mutations in PWS patients. As a highly expressed solute carrier protein in the cortex and the hippocampus, the increased mean precursor intensity of peptide from SLC25A5 was observed in AD, and the mutation of this gene was recognized as an important risk factor for a facial dysmorphism and seizures characterized by intellectual disability (ID) (11, 29). These lines of data suggest the crucial roles of *SLC25A5* mutation in regulating neuronal pathways. Importantly, another identified gene in this study, *KCNJ12*, also reported an increase in epileptic granule cells and regulated the dendritic excitability (30). Moreover, *KCNJ12* mutation affects the Kir ion channels-mediated neurovascular communication (31). Taken together, it is likely that these two mutations and other genetic alterations, such as *GNAQ*, *PI3K*, *AKT*, and *RASA1*, together contribute to the neuropathy in the progression of PWS, this is a matter of when the mutation was acquired.

In conclusion, our study identified two novel genetic mutations, *SLC25A5* and *KCNJ12*, in the sporadic PWS patients, and provided an understanding of PWS genetic determinants. The possible importance of these mutations in the pathogenesis of PWS needs to be further explored.

## Data Availability Statement

The datasets presented in this article are not readily available because of ethical/privacy restrictions. Requests to access the datasets should be directed to the corresponding author.

## Ethics Statement

The studies involving human participants were reviewed and approved by the Ethics Committee of Wuhan No. 1 Hospital affiliated to Tongji Medical College, Huazhong University of Science and Technology. Written informed consent to participate in this study was provided by the participants or their legal guardian/next of kin. Written informed consent was obtained from the individual(s) for the publication of any identifiable images or data included in this article.

## Author Contributions

L-QC and D-SL initiated and designed the study, Y-YH, L-LW, YX, QJ, and LS collected samples and validated clinical data. KC, S-SQ, and J-ZW performed the experiments and analyzed the data. KC and D-SL wrote the manuscript and revised it. All authors read and approved the final manuscript.

## Conflict of Interest

The authors declare that the research was conducted in the absence of any commercial or financial relationships that could be construed as a potential conflict of interest.

## Publisher’s Note

All claims expressed in this article are solely those of the authors and do not necessarily represent those of their affiliated organizations, or those of the publisher, the editors and the reviewers. Any product that may be evaluated in this article, or claim that may be made by its manufacturer, is not guaranteed or endorsed by the publisher.
